# Reciprocal Predictions Between Reading Achievement and Cognitive Flexibility Development in Children and the Mediating Roles of the Left Middle Frontal Gyrus

**DOI:** 10.1002/hbm.70309

**Published:** 2025-09-05

**Authors:** Leilei Ma, Yanpei Wang, Jiali Wang, Rui Chen, Gai Zhao, Zhiying Pan, Ningyu Liu, Haibo Zhang, Weiwei Men, Shuping Tan, Jia‐Hong Gao, Shaozheng Qin, Yong He, Qi Dong, Sha Tao

**Affiliations:** ^1^ State Key Laboratory of Cognitive Neuroscience and Learning Beijing Normal University Beijing China; ^2^ IDG/McGovern Institute for Brain Research Beijing Normal University Beijing China; ^3^ Center for MRI Research, Academy for Advanced Interdisciplinary Studies Peking University Beijing China; ^4^ Psychiatry Research Center, Beijing Huilongguan Hospital Peking University Beijing China

**Keywords:** cognitive flexibility, left middle frontal gyrus, reading achievement, salience network, school‐aged children

## Abstract

The development of reading skills and cognitive flexibility is crucial for success in childhood and adulthood. Although previous studies demonstrate the existing links between the development of cognitive flexibility and the reading acquisition in children, it remains unclear how baseline reading achievement influences later cognitive flexibility, or vice versa, particularly in relation to the underlying brain development. Therefore, in this prospective longitudinal study, we investigated the reciprocal prediction between reading achievement and cognitive flexibility, along with the underlying brain development that potentially mediated this relationship in school‐aged children. By employing a self‐recruited longitudinal dataset with two time points spaced 12 months apart, we found a significant association between baseline reading achievement and later cognitive flexibility, as well as between baseline cognitive flexibility and later reading achievement. Moreover, the left middle frontal gyrus emerged as a central neural hub supporting the development of both abilities. Increases in its gray matter volume and enhanced its functional connectivity to the salience network significantly mediated the longitudinal associations between reading achievement and cognitive flexibility. Taken together, our findings demonstrate the vital role of the left middle frontal gyrus in integrating language information processing and higher‐order cognitive control. This provides evidence for future reading or cognitive interventions.

## Graphical abstract

Learning to read may promote children's cognitive flexibility. Left middle frontal and its functional connectivity to anterior cingulate gyrus or insular may mediate the relationship between children's reading and cognitive flexibility.

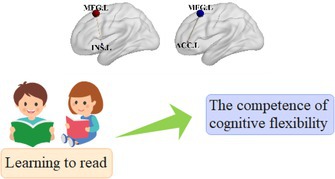

## 1 Introduction

Reading constitutes a higher‐order and complex cognitive skill, serving as a fundamental prerequisite for all other academic competencies (National Reading Panel 2000). It encompasses multiple components, including word decoding and reading comprehension (Ho et al. 2017). The Programme for International Student Assessment (PISA) defines reading as “the capacity to understand, use, reflect on and engage with written texts, enabling individuals to achieve their goals, develop their knowledge and potential, and participate effectively in society” (Eklund et al. 2018). Moreover, efficient and in‐depth reading processes, particularly when processing complex texts, engaging in reasoning, and achieving deep comprehension, heavily depend on an individual's cognitive flexibility. Cognitive flexibility involves the ability to shift perspectives, switch attention between tasks, and modify strategies to adapt to changing demands (Miyake et al. 2000). It is important for lifelong learning, problem‐solving, and the mental health of individuals (Buttelmann and Karbach 2017). Both cognitive flexibility and reading require engaging multiple cognitive processes to comprehend and adapt to environmental demands, and these capacities are critical for the developmental trajectories of school‐aged children.

Previous research has consistently identified significant associations between cognitive flexibility and reading performance across developmental stages. Cartwright's research demonstrates that children's cognitive flexibility predicts their reading comprehension performance (Cartwright and Kelly 2002). A growing body of research has subsequently validated the predictive role of cognitive flexibility in multiple reading‐related skills, including word/pseudoword decoding (Colé et al. 2014), reading fluency (Cartwright et al. 2019), oral language proficiency (Spencer et al. 2020), and reading comprehension (Johann et al. 2020; Hung and Loh 2021; Cantin et al. 2016; Fuhs et al. 2014; Kieffer et al. 2013). However, previous research has primarily focused on preschool or early elementary grades, lacking investigation into the rapidly developing school‐age period. Furthermore, cognitive performance during the school‐age years is closely associated with reading achievement. Reading achievement, encompassing word recognition, sentence comprehension, and discourse comprehension, aligns more closely with educational practice. The developmental trajectory of the relationship between reading achievement and cognitive flexibility during the school‐age period requires further investigation. Therefore, we aim to utilize longitudinal data to clarify the developmental relationship between reading achievement and cognitive flexibility in school‐age children and to provide a theoretical foundation for future evidence‐based educational practices.

Although cognitive flexibility and reading are associated, the underlying neural mechanisms remain unexplored. Functional magnetic resonance imaging revealed that children with dyslexia showed increased activity in multiple brain areas after language training (Temple et al. 2003). Given this finding, it is plausible that during critical neurodevelopmental periods in school‐aged children, prolonged systematic education may not only shape cognitive and reading competencies (Peng and Kievit 2020) but also induce neuroplastic adaptations in brain architecture. On the one hand, as children learn to read, their brains may undergo significant anatomical and functional changes. Researchers have reported that the inferior frontal gyrus (IFG), middle frontal gyrus (MFG), inferior parietal lobe (IPL), fusiform gyrus, temporal–occipital lobe, anterior cingulate cortex (ACC), and insula are important for reading (Siok et al. 2004, 2008, 2009; Tan et al. 2005; Benischek et al. 2020; Li and Bi 2022). On the other hand, task‐switching studies have also found the IFG, MFG, and temporoparietal junction regions (Sekutowicz et al. 2016; Uddin 2021; Worringer et al. 2019). Here we inquire: given that reading and cognitive flexibility may share rule representations and switching abilities to adapt to changing demands, do they also rely on shared brain regions for their regulation? Based on our review of the literature, we propose that both processes may converge on the MFG, IFG, and IPL.

Although structural neuroimaging reveals essential anatomical substrates for cognitive processes, the dynamic functional interactions between these regions may further elucidate the neural mechanisms underlying complex behaviors. Resting‐state functional connectivity (RSFC), which measures spontaneous low‐frequency fluctuations in neural activity during nontask conditions (Biswal et al. 1995; Fox and Raichle 2007), provides a critical lens to examine intrinsic brain network organization independent of task demands. Previous studies on reading have revealed enhanced functional connectivity within the insular network, as well as between the IFG and middle/superior temporal gyri in individuals with higher reading abilities (Chang et al. 2018). Comparative studies indicate that typically developing children exhibit stronger connectivity within the salience network relative to children with reading difficulties (Twait et al. 2018). Furthermore, a synthesis of empirical evidence demonstrates that the left MFG critically integrates ventral (occipital‐temporal regions) and dorsal (inferior parietal lobule/frontal gyrus) reading pathways (Guo et al. 2022). On the other hand, cognitive flexibility involves the cingulo‐insular network, which mediates switching between the medial and lateral frontoparietal networks to adapt to changing environmental demands (Kupis et al. 2021). Notably, Uddin's research has emphasized the crucial role of the salience network in cognitive flexibility (Dajani and Uddin 2015; Uddin 2015). Here we demonstrate the implication of the salience network in both reading and cognitive flexibility processes. This raises two key questions: Are these shared functional networks associated with their underlying shared structural substrates? Is their relationship mediated by structural‐functional brain connectivity? Synthesizing the literature, we propose that reading and cognitive flexibility engage convergent neural mechanisms within the salience network, exhibiting robust connectivity with their shared structural correlates. Critically, this integrated architecture may mediate the interplay between these cognitive domains.

Therefore, in this study, we first analyzed longitudinal tracking data from children aged 6 to 12 years to clarify the relationship between reading achievement and cognitive flexibility at the behavioral level. Next, we employed voxel‐based morphometry (VBM) to identify brain structural correlates of reading achievement and cognitive flexibility, delineating shared neural substrates between these abilities. We then examined the mediating role of these common structural features in their behavioral relationship to establish their mechanistic significance. Subsequently, using these shared regions as seed points, we conducted whole‐brain resting‐state functional connectivity analyses to uncover functional networks jointly associated with reading achievement and cognitive flexibility. This approach allowed us to further probe the intrinsic mechanisms underlying their interaction. Through integrated behavioral and neuroimaging evidence, we ultimately elucidate the shared mechanisms of rule representation and cognitive transformation that subserve both abilities.

## 2 Methods

### 2.1 Participants

Neuroimaging datasets from 343 children were obtained after quality control and two‐year matching from the Children School Functions and Brain Development Project (CBD, Beijing Cohort). CBD is an ongoing prospective longitudinal imaging cohort study of 6‐ to 12‐year‐old Chinese children. The imaging data and cognitive assessments were collected every year. Children were recruited from dozens of Beijing elementary schools. Parents/guardians signed the informed consent, and the children provided verbal consent. The exclusion criteria were as follows: (1) a history of neurological/psychiatric disorders (e.g., stroke, bipolar disorder), (2) a history of psychoactive drug use, (3) severe head injuries, or (4) diseases that contraindicate MRI use. A total of 355 children were examined over 2 years of behavioral data, of which 8 were excluded because the structural image quality was not satisfactory and 4 were not included in the brain scans for other reasons. The study protocol was reviewed and approved by the Ethics Committee of Beijing Normal University and conducted in accordance with the Declaration of Helsinki. Participant information is provided in Table 1.

**TABLE 1:** The summary demographic information of participants.

	Baseline (*n* = 343)	Follow‐up (*n* = 343)	*t* value
Age in years (mean ± SD)	8.96 ± 1.34	10.13 ± 1.41	
Sex: females, *n* (%)	155 (45.19%)		
Parental education (mean ± SD)^a^	8.66 ± 2.25		
Family income (mean ± SD)^a^	7.65 ± 1.58		
Reading achievements (mean ± SD)	522.73 ± 97.80	536.39 ± 106.17	2.45*
Cognitive flexibility (mean ± SD)	5.90 ± 2.07	6.87 ± 1.83	8.40***

^a^ The detailed information was presented in supplemental material.

* *p* < 0.05.

*** *p* < 0.001.

### 2.2 Reading Achievement and Cognitive Flexibility Evaluations

#### 2.2.1 Reading Achievement Test

The reading achievement test was based on the national curriculum and was developed by the National Children's Study of China (NCSC) project team (Dong 2011). The test evaluates character and word recognition as well as sentence and short‐passage comprehension. A representative national sample was standardized based on a normative sample of 28,800 school‐aged children whose Cronbach's α ranged from 0.75 to 0.89. This test only offers a total score without any subtest scores. The children completed the test individually in 45 min under the supervision of a professionally trained examiner.

#### 2.2.2 Cognitive Flexibility Test

The Wisconsin Card Sorting Task (WCST) was used to measure cognitive flexibility. In the WCST paradigm, children are required to match cards based on the number, shape, and color of the geometric objects that are printed on the cards. Children should independently discover the sorting rule based on feedback provided by the computer. Following 10 correct sorts, the sorting rule is changed without warning, and children must again discover the new sorting rule based on computer feedback (Cepeda et al. 2001). Previous studies have included three common indices: The number of categories switched, the total number of correct responses, and perseverative errors (Miyake et al. 2000). However, the use of perseverative errors as a measure of cognitive flexibility is limited, as it may not capture subtle differences between individuals (Reimers and Maylor 2005). Although a sensitivity index was proposed previously (Rhodes 2004), it is unable to account for individual differences in categorical data. Thus, we developed a new index to capture individual variance by combining the number of switches and correct responses. This new cognitive flexibility index is a continuous variable and provides a more accurate reflection of individual differences. The Cronbach's *α* was 0.986. The formula is as follows:

$$\text{Cognitive flexibility}=\text{Switches}+\frac{\mathrm{CR}-{\mathrm{CR}}_{\min\left(\text{within group}\right)}}{{\mathrm{CR}}_{\max\left(\text{within group}\right)}-{\mathrm{CR}}_{\min\left(\text{within group}\right)}+1}$$

where the switches is the number of times the child successfully switched the rule, and CR represents the number of correct responses. Children were grouped based on the number of switches, and within each group, correct responses were sorted. Each group had both the maximum and minimum number of correct responses. A new value was calculated for each participant using this formula.

### 2.3 Image Acquisition

The MR images were acquired at three sites: Peking University, Beijing HuiLongGuan Hospital, and Beijing Normal University, using a 3T Siemens Prisma scanner with the same imaging sequences. The high‐resolution T1‐weighted magnetization‐prepared rapid acquisition gradient echo (MPRAGE) parameters were as follows: repetition time = 2530 ms, echo time (TE) = 2.98 ms, field of view (FOV) = 256 × 224 mm^2^, effective voxel resolution = 1 × 1 × 1 mm, slice thickness = 1 mm, and number of slices = 192. The structural image was normalized to the standard atlas space. The scanning time of the structural image was 5 min and 58 s. The resting‐state fMRI used an echo–planar imaging (EPI) sequence with 240 volumes. It was acquired by multislice and single‐shot methods. The parameters were as follows: TR = 2000 ms, TE = 30 ms, flip angle = 90°, FOV = 224 × 224 mm^2^, matrix = 64 × 64, slice thickness = 3.5 mm, slices = 33, and voxel size = 3.5 × 3.5 × 3.5 mm^3^. In the scanner, the participants were instructed to stay still, keep their eyes open, and stare at the black screen in front of them without thinking about anything in particular. The resting‐state imaging process took 8 min and 6 s. To minimize children's head motion, a well‐trained researcher led the children through a practice scanning experience.

### 2.4 MRI Quality Control

We used international standardized quality control rules to examine the quality of MR images. The follow‐up brain data were included in the analysis. The processes were as follows: First, the radiologist carefully screened individual images and excluded those with incidental abnormalities, such as arachnoid cysts, neuroepithelial cysts, and other intracranial lesions. Second, five experienced raters evaluated each of the scanned images. The specific scan rating procedure was similar to the protocol used in the Human Connectome Project (Marcus et al. 2013). Third, all the brain images that passed the quality control criteria were retained. Finally, we applied framewise displacement (FD) as the criterion to check the quality of the resting‐state fMRI images (Power et al. 2012), in which functional images with a mean FD exceeding 0.5 mm were excluded from the samples (Xia et al. 2018).

### 2.5 Image Analysis Preprocessing

We used SPM12 software (http://www.fil.ion.ucl.ac.uk/spm) to segment the images. Based on the Computational Anatomy Toolbox (CAT12) embedded in SPM12 (http://dbm.neuro.uni‐jena.de/cat), we conducted voxel‐based morphometry (VBM). First, CAT12 default settings were used to segment the T1‐weighted images into gray matter, white matter, and cerebrospinal fluid. Then, we applied spatial normalization (voxel size of 2 mm^3^) to the gray matter images by Jacobian determinants. Next, the images were normalized to Montreal Neurological Institute space using the DARTEL approach. The individual gray matter volume was smoothed with a 4‐mm full width at half maximum Gaussian kernel. In the last step, we estimated the total intracranial volume (TIV), which was considered a covariate in subsequent analyses.

For the resting‐state fMRI data, we referred to Yan's DPABI software preprocessing procedures (http://rfmri.org) (Yan et al. 2016). The preprocessing steps were as follows: (1) head–motion correction, (2) slice–timing correction, (3) spatial normalization, (4) regressing out whole‐brain and white matter signals and 24 motion parameters, (5) spatial smoothing with a 6‐mm 3D full‐width at half‐maximum Gaussian kernel, and (6) temporal bandpass filtering (0.01–0.1 Hz).

### 2.6 Statistical Analysis

We constructed cross‐lagged panel models (CLPMs) in Mplus 7 to investigate the association between reading and cognitive flexibility. The scores of the behavioral data were age‐standardized. For the CLPM at the whole level, we set baseline reading achievement and cognitive flexibility as independent variables and those values at follow‐up as dependent variables. In the models, we controlled for demographic variables, including sex, parental education, family income, and the scanner site. Additionally, we explored the relationship between reading and cognitive flexibility based on demographic variables and ability levels. We standardized reading and cognitive flexibility separately, designating those with values greater than zero as the “higher” group and those with values less than zero as the “lower” group. We then used the continuous data for the subsequent analysis. In addition, we used DPABI software (http://rfmri.org) to analyze the MRI data (Yan et al. 2016). Pearson's correlation between gray matter volume and behavioral data was computed to acquire brain–behavior correlations. We used multiple comparison correction, with the significance threshold set at a voxel‐size value of *p* < 0.001 and a family‐wise error‐corrected cluster probability of *p* < 0.05. We subsequently identified the region that overlapped between reading and cognitive flexibility. Additionally, brain–behavior correlation analyses were conducted using SPSS 21 (IBM), and the mediating role of the brain area was determined. The shared region was turned into a mask, and the seed mask was used to calculate the whole‐brain functional connectivity. Finally, we explored the connectivity of this region and compared it between engaging in reading and cognitive flexibility using SPSS software. Although analyzing the data, we controlled for age, sex, scanner site, parental education, family income, FD, and TIV.

## 3 Results

The baseline and follow‐up characteristics of the study participants are presented in Table 1, with more detailed information in Tables S1–S3. Our results revealed that girls demonstrated greater reading achievement than boys at both baseline and follow‐up. Furthermore, cognitive flexibility in boys was lower than in girls at follow‐up, although no significant difference was observed at baseline (see Table S1). Parental education was found to influence reading achievement but not cognitive flexibility. A higher parental education level resulted in better reading achievement (Table S2). Family income level affected reading achievement at follow‐up (Table S3).

### 3.1 Reciprocal Predictions Between the Development of Reading Achievement and Cognitive Flexibility

**FIGURE 1:**
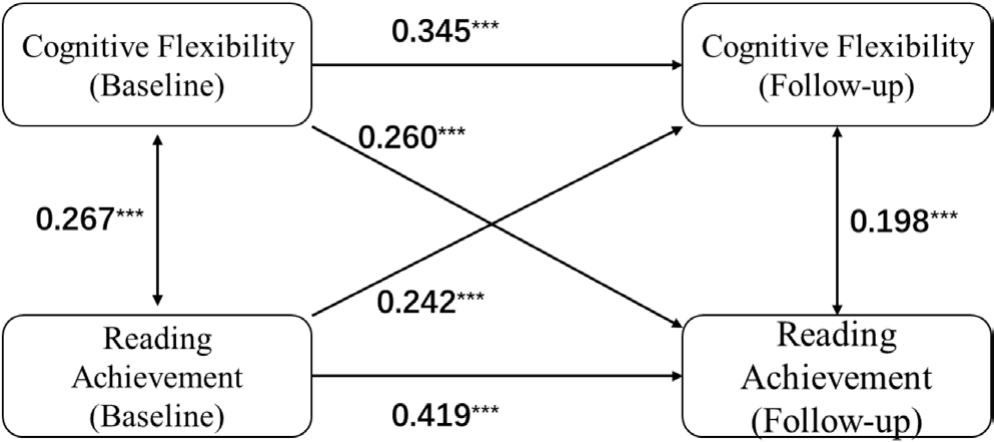
The predictions between reading achievement and cognitive flexibility in the cross‐lagged panel model. *p < 0.05, **p < 0.01, ***p < 0.001.

Children's reading achievement and cognitive flexibility were significantly correlated at both baseline and follow‐up (see Table S4). We applied the CLPM to explore the relationship between reading achievement and cognitive flexibility. The results indicated reciprocal predictions between reading achievement and cognitive flexibility development. Higher reading achievement at baseline predicted greater cognitive flexibility at the follow‐up evaluation (*β* = 0.242, *p* < 0.001; Figure 1). Similarly, higher baseline cognitive flexibility significantly predicted better reading achievement 1 year later (*β* = 0.260, *p* < 0.001; Figure 1).

To further determine the stable relationship between cognitive flexibility and reading achievement, a series of CLPM analyses were conducted to compare groups according to age, sex, parental education, family income, reading achievement levels, and cognitive flexibility levels. Both the age and sex groups showed bidirectional relationships between reading achievement and cognitive flexibility (Figures S1 and S2). Parental education and family income level did not moderate the relationship between reading achievement and cognitive flexibility (Figures S3 and S4). The reading achievement level did not significantly moderate the relationship (*χ* ^*2*^ = 0.250, *p* = 0.617; Figure S5). In addition, we observed a discrepancy in the association between baseline cognitive flexibility and subsequent reading achievement between the two groups (lower vs. higher cognitive flexibility). However, there were no moderating effects on the relationship between baseline cognitive flexibility and follow‐up reading achievement (*χ* ^*2*^ = 1.194, *p* = 0.275) or vice versa (*χ* ^*2*^ = 0.329, *p* = 0.566) (Figure S6). Only higher levels of cognitive flexibility promoted reading achievement at follow‐up, not lower levels of cognitive flexibility. Thus, cognitive flexibility was not a restrictive factor for reading achievement, but well‐developed cognitive flexibility could improve reading achievement. In contrast, reading achievement significantly contributed to the development of cognitive flexibility, regardless of whether the reading achievement level was lower or higher.

### 3.2 Brain Regions Associated With Reading Achievement and Cognitive Flexibility

**FIGURE 2:**
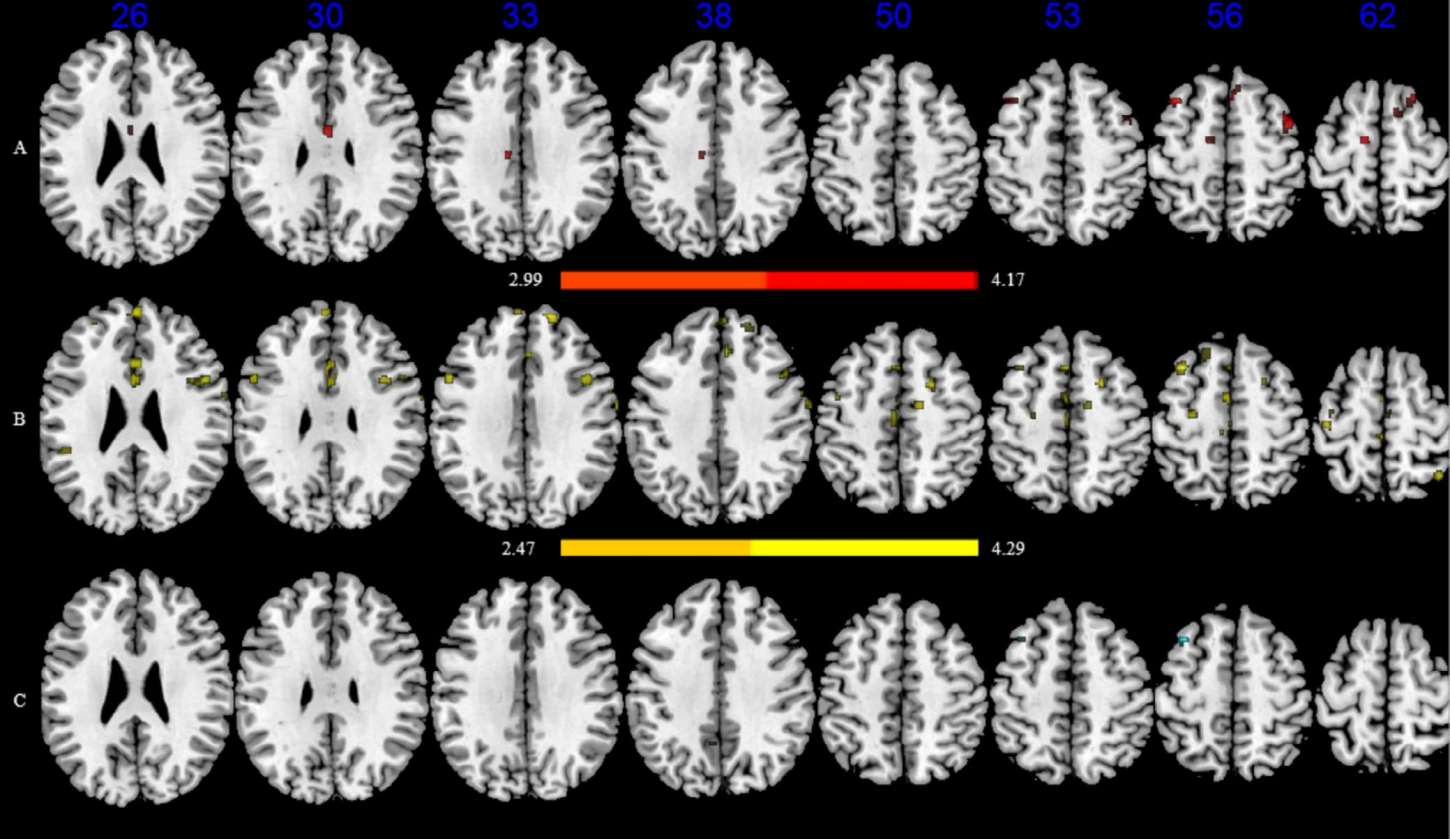
Brain regions associated with activation of reading achievement and cognitive flexibility. (A) Reading achievement involved significant brain areas in red. (B) Cognitive flexibility involved significant brain areas in yellow. (C) Brain areas significantly were associated with reading achievement and cognitive flexibility in cyan. The colour bar represents the *z* value.

We conducted a voxel‐based morphometry (VBM) analysis to identify the brain areas associated with reading achievement and cognitive flexibility. The results showed that reading achievement was associated with greater gray matter volumes in the middle frontal gyrus (MFG), superior frontal gyrus, parahippocampal gyrus, fusiform gyrus, middle/inferior occipital gyrus, thalamus, and cerebellum (Table S5, Figure 2A). Higher cognitive flexibility was correlated with greater gray matter volumes in the anterior cingulate gyrus, inferior/middle frontal gyrus, precentral gyrus, and insula (Table S6, Figure 2B). More importantly, the left MFG (27 voxels, peak MNI coordinates: −34, 18, 54) was identified as the neural region supporting the development of both reading achievement and cognitive flexibility (Figure 2C).

**FIGURE 3:**
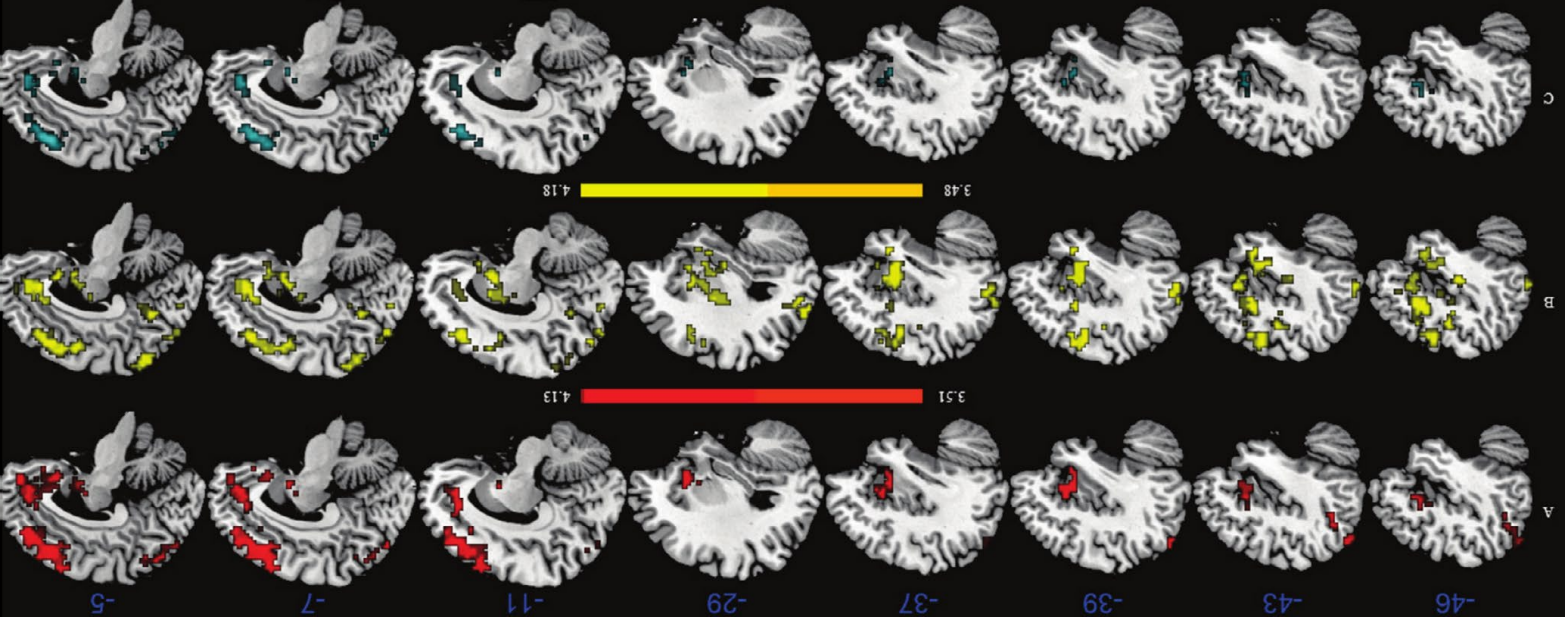
Whole brain functional connectivity in the left middle gyrus upon reading achievement and cognitive flexibility. (A) Reading achievement involved significant brain areas in red. (B) Cognitive flexibility involved significant brain areas in yellow. (C) Brain areas significantly were associated with reading achievement and cognitive flexibility shared in cyan (overlap in the left anterior cingulate cortex and insular lobule).

In addition, we investigated whether the left MFG regulated functional connectivity with the salience network, which influences the relationship between the development of reading achievement and cognitive flexibility. First, the left MFG was set as a seed region mask. The seed region mask was then set as the region of interest (ROI) to calculate the whole‐brain functional connectivity. In terms of reading achievement, results showed significantly increased functional connectivity of the left MFG with the left anterior cingulate cortex (ACC), insula, superior frontal gyrus (SFG), angular gyrus (AG), and inferior frontal gyrus (IFG) (Table S7, Figure 3A). In terms of cognitive flexibility, there was significantly increased functional connectivity of the left MFG with the insula, ACC, and medial/superior frontal gyrus (Table S8, Figure 3B). Spatial correlation analysis between reading achievement‐related functional connectivity maps and cognitive flexibility maps revealed shared neural substrates in the left ACC (142 voxels) and insular lobule (141 voxels) (Figure 3C).

### 3.3 The Mediating Effect of the Left MFG (Volume and Regulation of the Salience Network) on the Relationship Between Reading Achievement and Cognitive Flexibility

**FIGURE 4:**
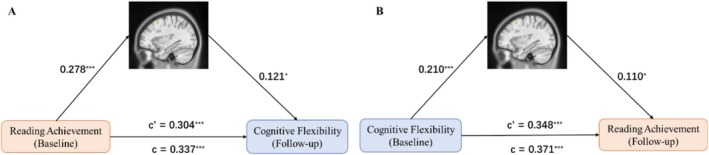
The mediated role of the left MFG between reading achievement and cognitive flexibility. (A) Reading achievement promoted the gray matter volume of the left MFG to predict cognitive flexibility development (indirect effect, 95% CI = [0.0072, 0.0699]). (B) Cognitive flexibility promoted the gray matter volume of the left MFG to predict reading achievement development (indirect effect, 95% CI = [0.0033, 0.0541]). *p < 0.05, **p < 0.01, ***p < 0.001.

The cross‐lagged analysis of reading achievement and cognitive flexibility in all participants indicated that they predict each other. Consequently, we examined the interrelationships of their shared structural and functional connectivity within the brain. Reading achievement may influence cognitive flexibility through modulating gray matter volume in the left MFG (Figure 4A); conversely, cognitive flexibility may reciprocally affect reading achievement via structural plasticity in the left MFG (Figure 4B). The left MFG serves as a neural mediator in this bidirectional relationship.

**FIGURE 5:**
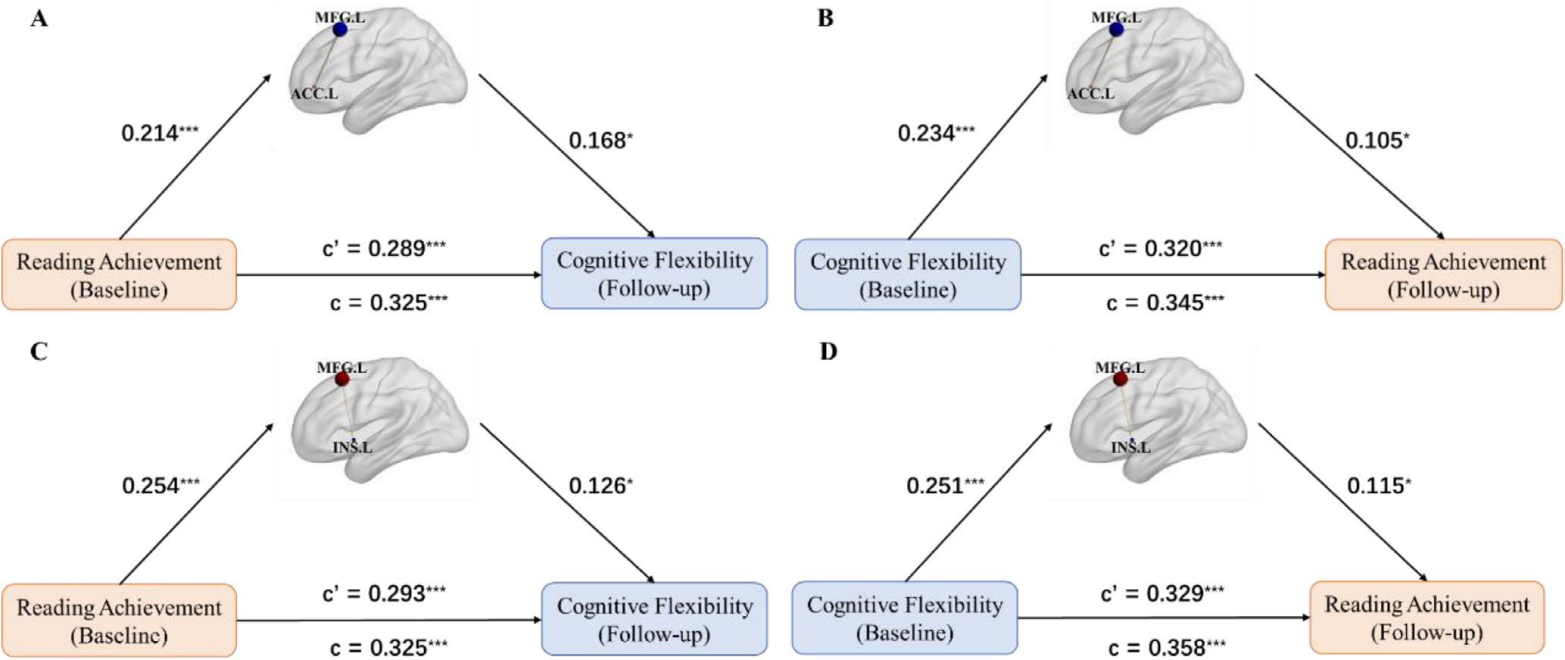
The mediated role of functional connectivity between left MFG and salience network on the prediction relation between reading achievement and cognitive flexibility. (A) Reading achievement enhanced the functional connectivity between left MFG and ACC to influence cognitive flexibility development (indirect effect, 95% CI = [0.0129, 0.0701]). (B) Cognitive flexibility enhanced the functional connectivity between left MFG and ACC to influence reading achievement development (indirect effect, 95% CI = [0.0072, 0.0691]). (C) Reading achievement enhanced the functional connectivity between left MFG and insula to influence cognitive flexibility development (indirect effect, 95% CI = [0.0059, 0.0686]). (D) Cognitive flexibility enhanced the functional connectivity between left MFG and insula to influence reading achievement development (indirect effect, 95% CI = [0.0071, 0.0682]). *p < 0.05, **p < 0.01, ***p < 0.001.

Further dynamic connectivity analysis revealed that the left MFG regulated the salience network (the ACC and insula). Reading achievement can influence the development of cognitive flexibility by modulating the connectivity between the ACC and the left MFG (Figure 5A), and vice versa (Figure 5B). Similarly, reading achievement can influence the development of cognitive flexibility by modulating the connectivity between the insula and the left MFG (Figure 5C), and vice versa (Figure 5D). The connectivity between the left MFG and ACC/insula mediated the relationship between reading achievement and cognitive flexibility, indicating that the left MFG regulates the salience network and that its dynamic functional connectivity explains the variance in the relationship between reading achievement and cognitive flexibility.

## 4 Discussion

This longitudinal study in school‐aged Chinese children revealed reciprocal predictions between reading achievement and cognitive flexibility development at a holistic level. This bidirectional association remained stable regardless of demographic variables, such as sex, parental education, and family income. Specifically, the influence of cognitive flexibility on reading achievement was moderated by baseline cognitive flexibility, with higher levels of cognitive flexibility at baseline promoting later (i.e., follow‐up) reading achievement. In contrast, reading achievement significantly promoted the development of cognitive flexibility regardless of baseline reading ability. Furthermore, our findings confirmed that the left MFG is a shared region that supports both reading achievement and cognitive flexibility, providing empirical evidence that growth of the left MFG and strengthening of its regulation of the salience network mediate reciprocal predictions between reading and cognitive flexibility in a longitudinal manner.

In this study, we examined the reciprocal relationship between reading achievement and cognitive flexibility. Although lower cognitive flexibility at baseline did not predict reading achievement at follow‐up, higher cognitive flexibility at baseline did. This suggests that cognitive flexibility plays a critical role in reading achievement throughout development. Previous studies suggest that executive function is multidimensional instead of one unified construct (Miyake et al. 2000), and research has shown that basic cognitive abilities (e.g., working memory and attention inhibition) are instrumental in promoting reading in early childhood (Peng et al. 2018; Wang et al. 2022). Cognitive flexibility develops upon the foundation of attention inhibition and working memory, undergoing significant refinement during the school years (Diamond 2013). Although previous research in this developmental period has identified bidirectional predictions between executive functions and reading (Zhang and Peng 2023; Meixner et al. 2019), the specific role of cognitive flexibility within this relationship remains underexplored. However, as children grow, advanced cognitive abilities, such as cognitive flexibility, become more essential to reading achievement. By enabling readers to employ different attention strategies, such as rereading, skimming, and information search, cognitive flexibility can coordinate bottom‐up and top‐down processing to enhance reading (Kieffer et al. 2013). Previous studies have found a bidirectional relationship between reading and cognitive flexibility in kindergarten‐aged children (Fong and Ho 2022; Fuhs et al. 2014; Willoughby et al. 2019). These studies used a two‐dimensional card sorting task to measure cognitive flexibility, and their reading assessments included word decoding, vocabulary knowledge, and sentence reading fluency. Our study used the classic WCST with three rules and four dimensions of features, and reading achievement was assessed in terms of basic character, word recognition, and complex sentence and short‐passage comprehension. Moreover, we extended these findings on the relationship between reading achievement and cognitive flexibility to elementary school students. In contrast to prior findings, reading significantly promoted the development of cognitive flexibility, regardless of reading ability. A review highlighted the impact of education on the reciprocal association between cognitive function and the development of reading (Peng and Kievit 2020). Reading is a complex process and involves substantial cognitive engagement. For children in elementary school, systematic reading instruction not only enhances their own reading ability but also contributes to cognitive development, including cognitive flexibility (Ceci and Williams 1997).

Our study provides evidence of neuroanatomical and functional overlap between reading achievement and cognitive flexibility, manifested in both structural convergence within the left MFG and functional connectivity between the left MFG and the salience network (ACC and insula). However, no overlapping neural substrates were observed in the IFG or IPL between the two processes. Brain–behavior correlation analyses independently identified associations involving these regions (IFG/IPL) for each cognitive domain. Early studies focusing on brain structures in typically developing Chinese children and those with dyslexia revealed significant differences in the left MFG, a region responsible for coordinating and integrating multimodal information, such as orthographic, phonological, and semantic information (Siok et al. 2004, 2008; Tan et al. 2005). However, subsequent research comparing language activation patterns in adults across Spanish, English, Hebrew, and Chinese demonstrated similar neural activation profiles during reading tasks (Rueckl et al. 2015). The role of the left MFG in mediating cognitive flexibility and reading performance may reflect its domain‐general capacity for executive control of complex information streams rather than language‐specific processing. Nee suggested that the MFG plays a critical role in cognitive control, potentially predicting a domain‐general form of contextual control (Nee and D'Esposito 2017). In addition, research has highlighted the role of the MFG in encoding joint task predictions (Jiang et al. 2018), and a meta‐analysis showed that the left MFG was activated in a cognitive flexibility task (Worringer et al. 2019). Behavioral studies identified a correlation between children's reading ability and cognitive flexibility in English (Cartwright and Kelly 2002; Cartwright et al. 2010; Kieffer et al. 2013), Portuguese (Engel de Abreu et al. 2014), Dutch (Nouwens et al. 2016), Danish (Søndergaard Knudsen et al. 2018), and Chinese languages (Fong and Ho 2022; Hung and Loh 2021). However, in identifying their neurocognitive mechanisms, future research is needed to elucidate whether differences exist under distinct language systems.

Wang et al. (2022) reported that the significant longitudinal association between attention and reading was mediated by the left MFG and its functional connectivity to the ventral attention network. Our study extended this view to higher cognitive functions (i.e., cognitive flexibility) and found that the growth of the left MFG and strengthened regulation of the salience network mediated the longitudinal associations between reading and cognitive flexibility. Indeed, cognitive flexibility involves the salience network, including the anterior cingulate cortex (attention convergence and reward anticipation) and insula (information representation) (Dajani and Uddin 2015; Uddin 2015, 2021). Specifically, the anterior cingulate cortex, as part of the limbic system, supports the attention process and is also part of the cingulo‐opercular network, which regulates mental and emotional processes (Bush et al. 2000). Intrinsic brain dynamics of the cingulate‐insular network (salience network) contribute to flexible cognition and behavior across the lifespan (Kupis et al. 2021). Therefore, the anterior cingulate and insula may participate in the reading process. Recent studies have supported that reading involves the anterior cingulate cortex and anterior insula, which participate in attention processing (Benischek et al. 2020; Chang et al. 2018; Li and Bi 2022) and play an important role in syllable‐level reading processing throughout the lifespan (Siok et al. 2020). Our study further extends this line of research, supporting that the left MFG regulates the salience network, thereby influencing the relationship between reading and cognitive flexibility development and revealing the intrinsic brain functional connectivity mechanism. Reading is a complex process that requires attentional control, working memory, and cognitive flexibility to coordinate different resources to maintain current representations and update them in response to changing circumstances. We found that the left MFG is the common neural basis of reading and cognitive flexibility development, and we provided empirical evidence that the growth of the left MFG and strengthened regulation of the salience network by this region mediate the longitudinal associations between reading and cognitive flexibility. In conclusion, the left MFG and regions in the salience network are essential for reading and cognitive flexibility, and they further promote cognitive flexibility development as individuals learn to read.

## 5 Conclusion

Reading and cognitive flexibility exhibited reciprocal predictions in a longitudinal cohort study of school‐aged children. The left middle frontal gyrus (MFG) serves as a region shared by reading and cognitive flexibility. We provide empirical evidence that the growth of the left MFG and strengthened regulation of the salience network by this region mediate the longitudinal association between reading and cognitive flexibility. In summary, our results indicate that learning to read supports the development of cognitive flexibility by facilitating the development of brain structures and functions, and vice versa.

## 6 Limitations

This study examined the relationship between reading achievement and cognitive flexibility at both the behavioral and neural levels. The results showed that the left MFG plays an important role in regulating the salience network. However, this study has several limitations. We applied a series of CLPMs to investigate the longitudinal relationship between reading achievement and cognitive flexibility. We found a stable reciprocal association between reading achievement and cognitive flexibility across demographic characteristics (age, sex, parental education, and family income), except in the group with high or low cognitive flexibility at baseline. Because we used data from two time points, we were unable to identify the within‐variable and within‐time correlations. Future studies should examine a longer period, including three time points, to acquire a clearer predictive relationship between reading and cognitive flexibility. We used voxel‐based morphometry to explore the brain regions shared by reading and cognitive flexibility and computed the functional connectivity of these regions using resting‐state fMRI. These analyses revealed a critical pathway between the left MFG and the salience network. However, task‐based fMRI analysis might reveal a direct common region between reading and cognitive flexibility. This study utilized reading achievement scores. Future research may focus on the relationships between different components of reading and cognitive flexibility, thereby providing a clearer mechanism underlying their interactions. The study did not compare the relationships in different language systems. This warrants further exploration.

## Author Contributions

Sha Tao, Yanpei Wang, and Leilei Ma conceived and designed the study. Leilei Ma, Yanpei Wang, Jiali Wang, Rui Chen, Zhiying Pan, Gai Zhao, Haibo Zhang, and Ningyu Liu collected the data under the supervision of Sha Tao, Shuping Tan, Weiwei Men, and Jia‐Hong Gao. Leilei Ma performed data analysis under the supervision of Yanpei Wang and Sha Tao. Leilei Ma and Sha Tao wrote the manuscript. Leilei Ma, Sha Tao, and Yanpei Wang amended and proofread the manuscript. Sha Tao, Qi Dong, Shaozheng Qin, and Yong He designed the prospective study. Qi Dong and Sha Tao provided the fund. All authors reviewed and commented on the manuscript.

## Conflicts of Interest

Dr. Gao is a member of the HBM Editorial Board and co‐author of this article. To minimize bias, he was excluded from all editorial decision‐making related to the acceptance of this article for publication.

## Supporting information


**Data S1:** hbm70309‐sup‐0001‐Supplemental_Information.

## Data Availability

The raw data supporting the conclusions of this article were from the Children's School Functions and Brain Development Project (CBD, Beijing Cohort), which will be soon made public.
