# Supplementary material for: Reciprocal Predictions Between Reading Achievement and Cognitive Flexibility Development in Children and the Mediating Roles of the Left Middle Frontal Gyrus
Source: Hum Brain Mapp. 2025 Sep 5;46(13):e70309. doi: 10.1002/hbm.70309 (PMC12411736; doi:10.1002/hbm.70309)
Supplement: Supplementary file 1 — Data S1: hbm70309‐sup‐0001‐Supplemental_Information. [file HBM-46-e70309-s001.docx]

**Supplemental Information**

**The details of the parental education and family income are as follow:**

The parent's education level was indicated by the highest level of education achieved by parents. 1 = no schooling; 2 = Primary education; 3 = Junior high school; 4 = Senior high school; 5 = Secondary vocational school/Polytechnic school; 6 = Higher vocational education/Junior college (part-time); 7 = Junior college(full-time); 8= Bachelor degree (part-time); 9 = Bachelor degree (full-time); 10 = Graduate education or above.

Family Income (RMB/year): 1 = Less than 3000; 2 = 3001-6000;3 = 6001-10000;4 = 10001-30000; 5 = 30001-50000;6 = 50001-100000;7 = 100001-150000;8 = 150001-200000; 9 = Over 200001.

**Supplementary Table S1-S9**

Table S1 The sex differences of reading achievements and cognitive flexibility performance at baseline and follow-up.

|  | Baseline(*n* = 343) | | | Follow-up(*n* = 343) | | |
| --- | --- | --- | --- | --- | --- | --- |
|  | girls(n = 155) | boys(n = 188) | *t* value | girls(n = 155) | boys(n = 188) | *t* value |
| Reading achievements | 540.24±90.93 | 508.30±101.10 | -3.05^**^ | 553.57±102.41 | 522.22±107.39 | -2.75^*^ |
| Cognitive flexibility | 6.13±2.18 | 5.72±1.96 | -1.82 | 7.24±1.80 | 6.56±1.80 | -3.49^**^ |

*Note.* Descriptive statistics are reported as mean±SD. ^*^ *p*<0.05, ^**^ *p*<0.01, ^***^ *p*<0.001.

Table S2 The parental education differences of reading achievements and cognitive flexibility performance at baseline and follow-up.

|  | Baseline(*n* = 343) | | | Follow-up(*n* = 343) | | |
| --- | --- | --- | --- | --- | --- | --- |
|  | low(n = 178) | high(n = 165) | *t* value | low(n = 178) | high(n = 165) | *t* value |
| Reading achievements | 499.19±103.11 | 548.13±84.98 | 4.78^***^ | 515.18±112.01 | 559.27±94.61 | 3.92^***^ |
| Cognitive flexibility | 5.74±2.14 | 6.08±1.99 | 1.50 | 6.77±1.90 | 6.98±1.76 | 1.05 |

*Note.* High education means bachelor degree(full-time) or above. Descriptive statistics are reported as mean±SD. ^*^ *p*<0.05, ^**^ *p*<0.01, ^***^ *p*<0.001.

Table S3 The family income differences of reading achievements and cognitive flexibility performance at baseline and follow-up.

|  | Baseline(*n* = 343) | | | Follow-up(*n* = 343) | | |
| --- | --- | --- | --- | --- | --- | --- |
|  | low(n = 125) | high(n = 218) | *t* value | low(n = 125) | high(n = 218) | *t* value |
| Reading achievements | 511.63±98.53 | 529.09±97.03 | 1.60 | 515.05±111.30 | 548.62±101.36 | 2.85^**^ |
| Cognitive flexibility | 5.67±2.13 | 6.04±2.03 | 1.57 | 6.69±1.88 | 6.97±1.80 | 1.33 |

*Note.* High family income means more than 150000 RMB/year. Descriptive statistics are reported as mean±SD. ^*^ *p*<0.05, ^**^ *p*<0.01, ^***^ *p*<0.001.

Table S4 The correlation matrix between reading and Cognitive flexibility performance at baseline and follow-up.

|  |  | Cognitive flexibility | |
| --- | --- | --- | --- |
|  |  | BL | FU |
| Reading achievements | BL | 0.267^***^ | 0.334^***^ |
|  | FU | 0.372^***^ | 0.392^***^ |

*Note.* BL = baseline; FU = follow-up; ^*^ *p*<0.05, ^**^ *p*<0.01, ^***^ *p*<0.001.

Table S5 Significant GMVs associated with reading achievements.

| Region’s name | Voxels | MNI coordinates | | | z-score | laterality |
| --- | --- | --- | --- | --- | --- | --- |
|  |  | x | y | z |  |  |
| Cerebelum_9/Cerebellum Anterior/Posterior Lobe/Brainstem | 674 | -1.5 | -48 | -36 | 4.17 | Left |
| Parahippocampa Gyrus/Hippocampus | 200 | 30 | -16.5 | -24 | 3.51 | Right |
| Superior Frontal Gyrus | 55 | 6 | 60 | -22.5 | 2.99 | Right |
| Cerebelum_4_5/Fusiform | 86 | -13.5 | -40.5 | -18 | 3.24 | Left |
| Inferior Frontal Gyrus/Insula | 89 | 48 | 25.5 | -6 | -3.89 | Right |
| Thalamus | 271 | 10.5 | -28.5 | 6 | 3.39 | Right |
| Middle Temporal Gyrus/ Superior Temporal Gyrus | 476 | 54 | -69 | 10 | -3.33 | Right |
| Cingulate Gyrus | 466 | -3 | -46 | 36 | -3.14 | Left |
| Middle Occipital Gyrus/Superior Occipital Gyrus | 127 | -36 | -94.5 | 13.5 | -3.06 | Left |
| Middle Frontal Gyrus | 94 | -33 | 18 | 56 | 3.86 | Left |
| Anterior Cingulate/Limbic Lobe | 101 | 14 | 33 | 12 | 2.89 | Right |
| Parietal Lobe/ SupraMarginal/ Inferior Parietal Lobule | 121 | -51 | -29 | 24 | -3.39 | Left |
| Superior Occipital Gyrus | 383 | 16.5 | -94.5 | 31.5 | -3.92 | Right |
| Middle Frontal Gyrus | 84 | 42 | 0 | 57 | 3.40 | Right |

*Note.* Covariates: sex, age, parental education, family income, FD, TIV, and site. *p*<0.001 at voxel level and a cluster-level Family-wise Error (FWE) *p*<0.05 corrected.

Table S6 Significant GMVs associated with cognitive flexibility.

| Region’s name | Voxels | MNI coordinates | | | z-score | laterality |
| --- | --- | --- | --- | --- | --- | --- |
|  |  | x | y | z |  |  |
| Cerebelum_8/Cerebellum Anterior Lobe/Cerebellum Posterior Lobe | 182 | 22.5 | -58.5 | -37.5 | -3.73 | Right |
| Middle Occipital Gyrus | 58 | -43.5 | -78 | 6 | -3.03 | Left |
| Medial Frontal Gyrus/ Superior Frontal Gyrus | 210 | 3 | 56 | 26 | 3.37 | Right |
| Anterior Cingulate | 240 | -2 | 21 | 26 | 3.51 | Left |
| Inferior Frontal Gyrus/ Middle Frontal Gyrus | 187 | 46.5 | 13.5 | 37.5 | 3.51 | Right |
| Precentral Gyrus | 70 | -32 | -23 | 68 | 4.29 | Left |
| Middle Frontal Gyrus/ Inferior Frontal Gyrus | 51 | -27 | 50 | 21 | 3.27 | Left |
| Middle Occipital Gyrus/ Angular Gyrus/ Inferior Parietal Lobule | 119 | -30 | -60 | 36 | -3.36 | Left |
| Precentral Gyrus | 111 | -31.5 | -22.5 | 67.5 | 4.29 | Left |
| Precentral Gyrus | 102 | 36 | -16.5 | 67.5 | 3.58 | Right |
| Insula | 35 | -37.5 | 13.5 | 7.5 | 2.47 | Left |

*Note.* Covariates: sex, age, parental education, family income, FD, TIV, and site. *p*<0.001 at voxel level and a cluster-level Family-wise Error (FWE) *p*<0.05 corrected.

Table S7 Significant resting connections with left middle frontal gyrus associated with reading achievements.

| Region’s name | Voxels | MNI coordinates | | | z-score | laterality |
| --- | --- | --- | --- | --- | --- | --- |
|  |  | x | y | z |  |  |
| Inferior Frontal Gyrus/Superior Temporal Gyrus/Insula | 182 | 48 | 3 | -21 | 3.51 | Right |
| Insula/Inferior Frontal Gyrus/ Superior Temporal Gyrus | 263 | -30 | 15 | -12 | 3.76 | Left |
| Anterior Cingulate/Medial Frontal Gyrus | 305 | -6 | 21 | -12 | 3.77 | Left |
| Angular/Superior Temporal Gyrus/ Inferior Parietal Lobule | 151 | -54 | 66 | 36 | 3.53 | Left |
| Superior Frontal Gyrus/Medial Frontal Gyrus | 812 | -3 | 30 | 51 | 4.13 | Left |
| Precuneus/Superior Parietal Lobule | 202 | 27 | -72 | 54 | -3.26 | Right |
| Lingual Gyrus/Fusiform Gyrus | 128 | 21 | -84 | -15 | -3.39 | Right |

*Note.* Covariates: sex, age, parental education, family income, FD, and site. *p*<0.01 at voxel level and a cluster-level Family-wise Error (FWE) p<0.05 corrected.

Table S8 Significant resting connections with left middle frontal gyrus associated with cognitive flexibility.

| Region’s name | Voxels | MNI coordinates | | | z-score | laterality |
| --- | --- | --- | --- | --- | --- | --- |
|  |  | x | y | z |  |  |
| Left Cerebrum/Inferior Frontal Gyrus/Precentral Gyrus/Superior Temporal Gyrus/Parahippocampa Gyrus/Middle Temporal Gyrus/Thalamus/Fusiform/Insula | 3062 | 3 | 21 | -6 | 4.18 | Right |
| Precuneus/ Superior Parietal Lobule/ Superior Occipital Gyrus | 1544 | 27 | -75 | 54 | -4.27 | Right |
| Precentral Gyrus/ SupraMarginal/Inferior Frontal Gyrus/Inferior Parietal Lobule | 192 | 57 | 0 | 33 | 3.48 | Right |
| Cingulate Gyrus/ Superior Frontal Gyrus/ Medial Frontal Gyrus | 421 | -3 | 24 | 54 | 4.10 | Left |

*Note.* Covariates: sex, age, parental education, family income, FD, and site. *p*<0.01 at voxel level and a cluster-level Family-wise Error (FWE) p<0.05 corrected.

**Supplementary Figures S1-S6**


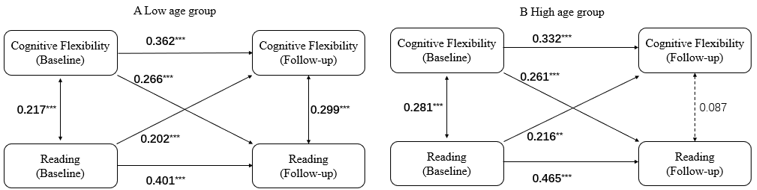


**Figure S1.** Cross-lagged analysis between cognitive flexibility and reading achievements in A) low and B) high age group.


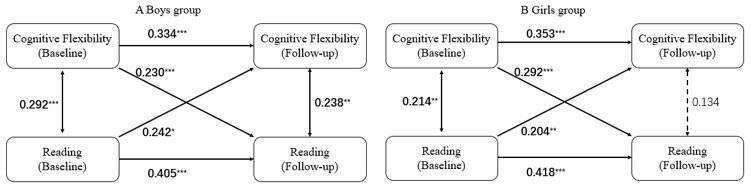


**Figure S2.** Cross-lagged analysis between cognitive flexibility and reading achievements in A) boys and B) girls age group.


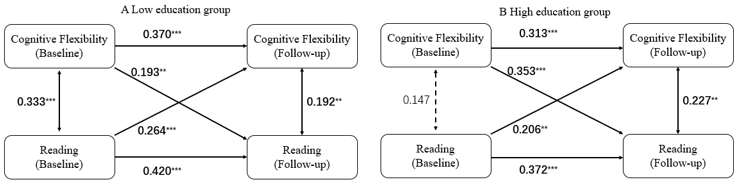


**Figure S3.** Cross-lagged analysis between cognitive flexibility and reading achievements in A) low and B) high education group.


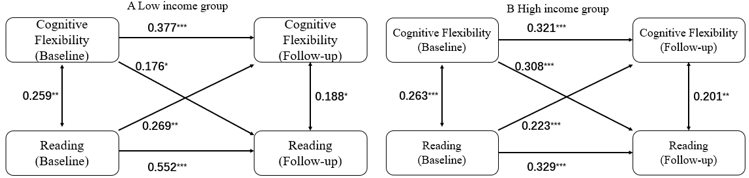


**Figure S4.** Cross-lagged analysis between cognitive flexibility and reading achievements in A) low and B) high income group.


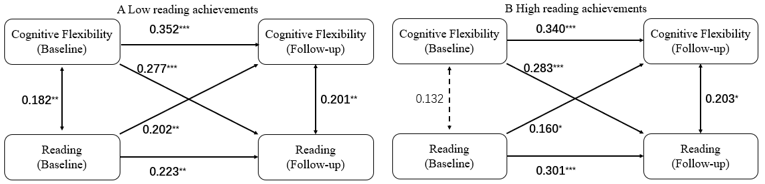


**Figure S5.** Cross-lagged analysis between cognitive flexibility and reading achievements in A) low and B) high reading achievements group.


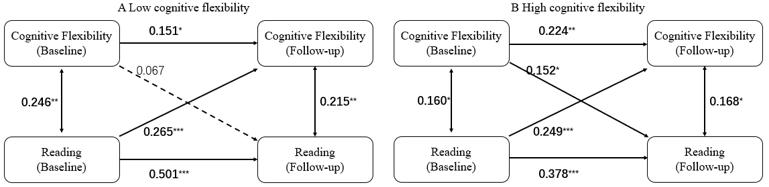


**Figure S6.** Cross-lagged analysis between cognitive flexibility and reading achievements in A) low and B) high cognitive flexibility group.
